# Pertussis vaccination in adults: a behavioral study of physicians from the US, France, and Germany

**DOI:** 10.1186/s12875-024-02647-3

**Published:** 2024-11-26

**Authors:** Donald Middleton, Liana Clark, Anne Mosnier, Ulrich Heininger

**Affiliations:** 1 University of Pittsburgh School of Medicine, Pittsburgh, PA USA; 2 Sanofi, Swiftwater, PA USA; 3 Open Rome, Paris, France; 4 University of Basel Children’s Hospital, Basel, Switzerland

**Keywords:** Bordetella pertussis, Whooping cough, Pertussis vaccine, Pertussis vaccination, Tdap, Physician recommendations, Physician behavior, Chronic illnesses, Attitudes, International

## Abstract

**Background:**

Pertussis is a highly contagious respiratory tract infection that affects all ages, though it is most severe in young infants. Adults, especially those with respiratory conditions or other chronic illnesses can also suffer serious consequences of pertussis. Pertussis vaccination is the best method of disease prevention in a lifetime. This behavioral study aimed to assess physicians’ attitudes towards pertussis vaccination in adults and the importance of pertussis vaccination for disease prevention, especially in those with chronic illnesses, and to determine the impact of the COVID-19 pandemic on adult vaccination behaviors.

**Methods:**

Between November 2022 and January 2023, physicians from the US, France, and Germany registered in an independent online database were contacted to participate in this study. After eligibility screening, participating physicians completed an online questionnaire addressing topics related to physician recommendations and vaccination behavior around pertussis in adults.

**Results:**

Eight hundred physicians participated in the study (US: 400; France: 200; Germany: 200). Physicians’ attitudes towards pertussis vaccination in adults were broadly similar between the countries. Overall, 65% of physicians believed in the importance of vaccination against pertussis, a lower proportion than for COVID-19 (82%), influenza (81%), pneumococcal disease (76%), and tetanus (73%). Physicians considered immunocompromised adults or those with chronic obstructive pulmonary disease (COPD), asthma, or other respiratory conditions to be at greatest risk from pertussis. Physicians estimated that two-thirds of the adult patients to whom they recommended pertussis vaccination agreed to receive it. The top reason why they felt patients did not receive pertussis vaccination as recommended was low perception of personal risk for pertussis. Physicians’ pertussis vaccination behavior was found to be similar before and after the COVID-19 pandemic.

**Conclusions:**

While physicians in the surveyed countries recognized the value of pertussis vaccination in adults, they ranked its importance lower than that of other adult vaccines. Physicians recognized the need to immunize vulnerable adults who are at risk of severe pertussis, such as those with asthma and/or COPD, but this awareness frequently did not result in vaccination of these priority groups, especially without official recommendations to support such vaccination in these groups.

**Supplementary Information:**

The online version contains supplementary material available at 10.1186/s12875-024-02647-3.

## Background

Pertussis, also known as whooping cough, is a highly contagious, serious infection of the respiratory tract predominantly caused by the bacterium *Bordetella pertussis*. This bacterium is transmitted from person to person, mainly through droplets produced during coughing and sneezing [1].

Prior to routine vaccination in childhood, pertussis was one of the most common diseases in children [2]. Currently, infants, especially those too young to be vaccinated, are at the highest risk of developing pertussis-associated complications [3]. As such, pertussis vaccination of pregnant people is recommended in many countries to provide passive immunity to the neonate.

However, in the focus on infant disease, there is sometimes a lack of recognition of the impact of pertussis disease in adults, especially those with chronic conditions. Adults with pertussis have similar symptoms to children with pertussis: paroxysmal cough (which can last for over three months), whoop after cough, and post-tussive emesis are the most common symptoms [2, 4]. They may also experience complications of pertussis such as sinusitis (13%), otitis media (4%), urinary incontinence (4%), pneumonia (4%), weight loss (3%), rib fracture (2%), and fainting (2%) [5].

The burden of pertussis is especially high for adults with respiratory conditions or other chronic illnesses and the elderly. Compared with the general population, adults with asthma are up to 4 times, and adults with COPD up to 2.5 times, more likely to be diagnosed with pertussis [6]. Several studies have shown that pertussis can acutely exacerbate asthma and chronic obstructive pulmonary disease (COPD) symptoms [6–11]. Those with asthma or COPD who contract pertussis are also up to 40% and 75%, respectively, more likely to be hospitalized than those without these conditions [6]. Moreover, a significant percentage of older people infected with *Bordetella pertussis* experience considerable morbidity, mortality, and costs [12].

Recently, we have seen a worrying spike in pertussis cases in several regions, including across Europe and the US [13, 14]. These cases include infants, children, teens, and adults. Since the best prevention of pertussis is for pertussis vaccination throughout the lifespan, adult vaccination is an important component of pertussis prevention and herd protection for communities [2, 15].

In many countries, public health vaccination strategies for pertussis prevention focus primarily on protecting infants and children, but less so for adults. For adults, national vaccination recommendations for pertussis immunization vary considerably by country, with most countries not having recommendations for the pertussis vaccination of non-pregnant adults. The US, France, and Germany all have recommendations for routine pertussis vaccination of adults. These recommendations are summarized in Supplementary Table 1. It should be noted, however, that there are also no specific official recommendations for adults at a higher risk of developing severe pertussis disease, e.g., those with asthma or COPD or the elderly, in these or any country. The US Centers for Disease Control and Prevention (CDC) suggests that those with lung disease receive vaccination against pertussis [16], but there is no official recommendation for this group beyond the general adult recommendation [17]. The result is inconsistent protection of adults across the lifespan, including those who are at higher risk from pertussis.

**Table 1:** Physician sample

	US	France	Germany
Number of physicians	400	200	200
Years in practice, mean	18	21	20
Specialty, n (%)			
General practitioner (France and Germany only)	–	200 (100)	200 (100)
Primary care practitioner (US only)	400 (100)	–	–
Family practitioner	162 (40.5)	–	–
Internist	214 (53.5)	–	–
Unspecified	24 (6.0)		
Setting, n (%)			
Public, outpatient practice or clinic	246 (61.5)	200 (100)	200 (100)
Private practice or clinic	154 (38.5)	0	0
Number of patients seen monthly, mean			
With asthma	43	42	27
With COPD	51	41	35
With other medical conditions	32	26	19
Aged 4–6 years	24	71	20
Aged 7–17 years	34	69	35
Aged 18–65 years	174	213	155
Aged > 65 years	141	162	142

COPD, chronic obstructive pulmonary disease

Physicians’ perception of the severity of pertussis in adults and its importance for vaccine prevention drives the strength of vaccination recommendation, which in turn impacts vaccine uptake [18]. In this international behavioral study of healthcare professionals, we sought to assess their perceptions of the risk of pertussis in adult patients and the importance of pertussis vaccination in this population, especially those with chronic medical conditions. Physicians’ behavior in recommending pertussis vaccination, facilitators and barriers to vaccination recommendation, and the impact of COVID-19 on physicians’ vaccination recommendation were also assessed.

## Methods

The study was conducted in the US, France, and Germany between November 30, 2022 and January 17, 2023. All recruitment and research were conducted in accordance with the European General Data Protection Regulation (GDPR) and the European Pharmaceutical Market Research Association (EphMRA) code of conduct.

### Physician sample

**Fig. 1:**
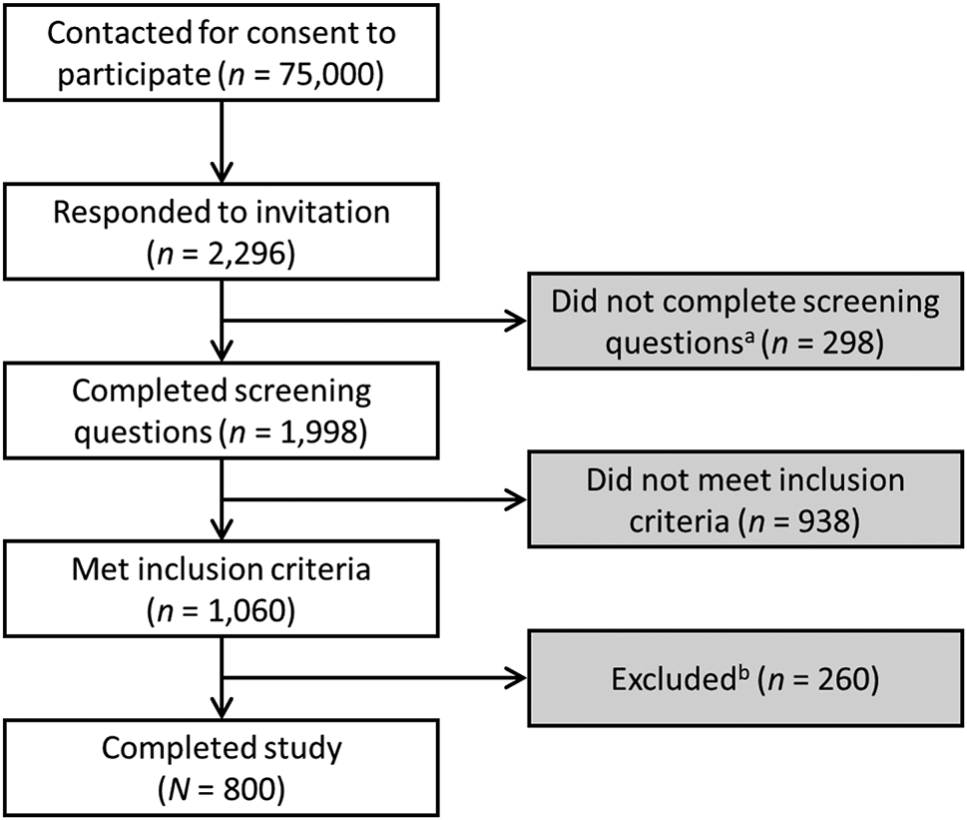
Physician flow chart. ^a^ Also includes physicians who were excluded during quality check. ^b^ Excluded from study as their specialty or country quota had already been fulfilled, or if they did not complete the questionnaire

The goal for this study was to recruit 800 physicians who provide care for adult patients from three countries (400 from the US, 200 from France, and 200 from Germany). Verified healthcare professionals who had previously expressed interest in participating in research activities were included in an independent online database, M3’s panel. Through this panel provider, 75,000 eligible physicians (including general practitioners [France and Germany], family practitioners and internists [US only]) were contacted via email for their consent to participate in the study (Fig. 1). A total of 2,296 physicians responded to the invitation, of whom 1,998 were assessed for whether they met the following inclusion criteria: (i) being personally involved in the decision to vaccinate adults and prescribe Tdap (or Tdap-IPV) vaccination, (ii) being in practice for more than 3 years and less than 44 years, and (iii) having a patient load consisting primarily of adult patients, including those with asthma and/or COPD. The full eligibility criteria are available as supplementary material (see Additional File 1]. Physicians recruited from the US also had to have a self-reported board certification in internal medicine or family practice. Nine hundred and thirty-eight physicians did not meet the eligibility criteria and, of the remaining 1,060 physicians, 260 were excluded from the study as their specialty or country quota had already been fulfilled, or if they did not complete the study. In accordance with GDPR and EphMRA codes of conduct, physician anonymity was maintained throughout the study.

The sample size was set at 800 to allow comparisons between countries at a 95% confidence interval with a margin of error of 3–7%, assuming sample proportion of 50% within a normally distributed dataset. Questionnaires were completed by 800 physicians across the US (*n* = 400), France (*n* = 200), and Germany (*n* = 200). Participating physicians were incentivized through Research Panel Credit (panel points that can be exchanged as cash paid to oneself or to a charity, or as gift cards).

### Physician questionnaire

The questionnaire consisted of three sections. Section A assessed physicians’ perception of pertussis disease in adults (four questions). Section B assessed physicians’ behavior in recommending pertussis vaccinations, their patients’ behaviors towards pertussis vaccinations, and the impact of the COVID-19 pandemic on vaccination behaviors (18 questions). Section C assessed topics relating to pertussis vaccine (such as vaccine efficacy, safety, co-administration, etc.), including preferred channels to garner this information (seven questions). Question formats included: multiple choice, open numeric (range 0–100), rating, and ranking. The full questionnaire can be found in the supplementary material (Additional File 1).

### Statistical analysis

Statistical tests were applied after data were tested for distribution and all relevant data aggregated. For dependent variables, t-tests for proportions or the analysis of variance test were used to analyze data from the same individuals. For independent variables, t-tests (for proportions), or Z-tests (for means) were applied to analyze test scores between different subgroups.

## Results

### Physician sample

The 800 participating physicians’ specialties are shown in Table 1. All physicians from France and Germany were general practitioners in public, outpatient practices, or clinics. In the US, 162 (40.5%) physicians were family practitioners and 214 (53.5%) were internists; 24 (6.0%) physicians did not specify their specialty.

### Section A

#### Physicians’ perceptions about pertussis vaccination

**Fig. 2:**
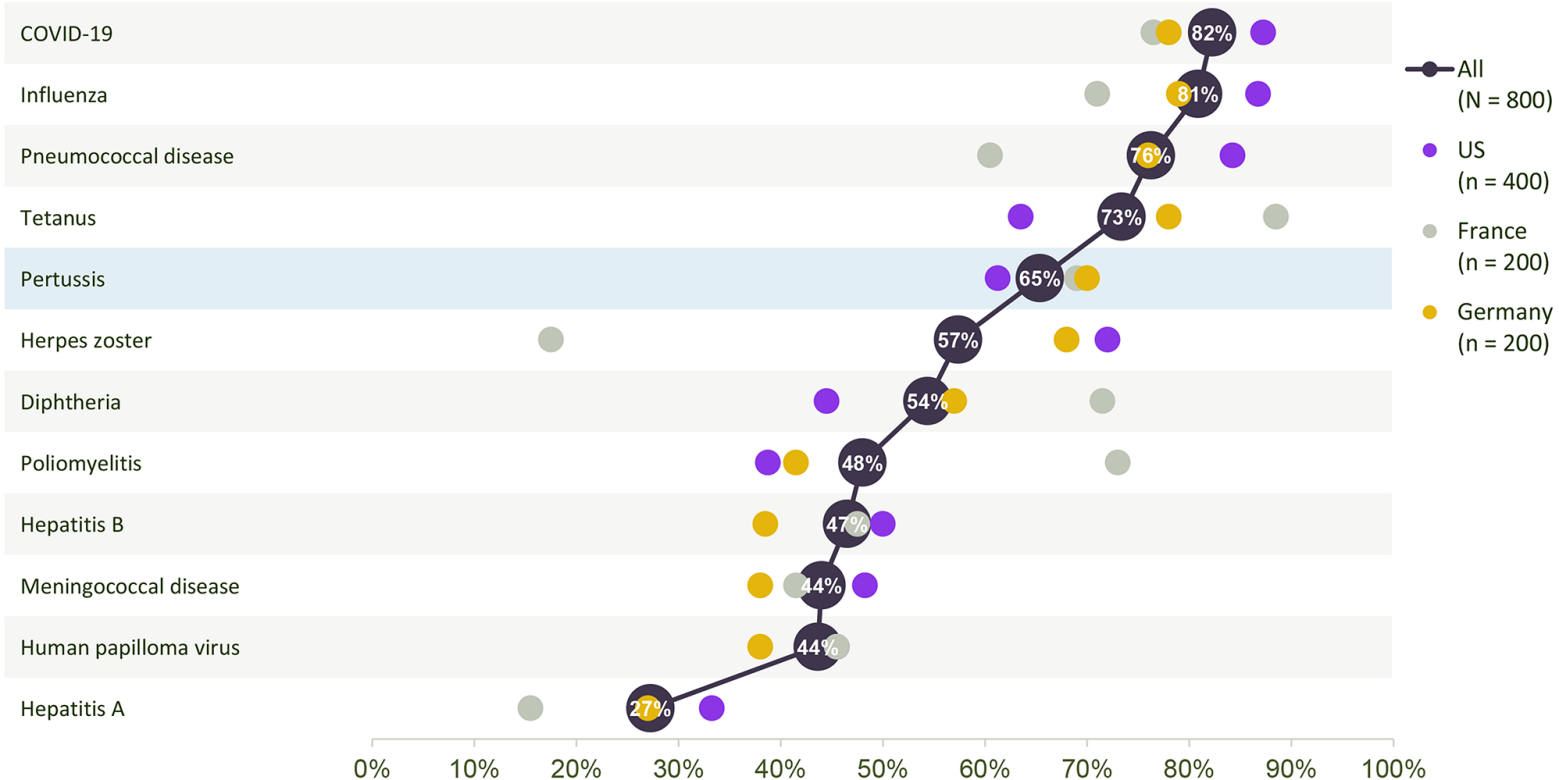
Physicians’ perception of diseases that require vaccination, ranked in order of need to vaccinate. Response to the study question ‘What is your perception of the need to vaccinate against the following diseases when thinking of your adult patients?’

Physicians’ perception of the importance of pertussis vaccination of adults was found to be similar between the three countries. Overall, 523 (65%) physicians believed in a need for universal pertussis vaccination in adults. Physicians’ perceived need to vaccinate against pertussis differed between the US, France, and Germany. The perceived importance of pertussis vaccination was most similar between France and Germany (*p* = 0.829). When compared with data from US physicians, US physicians perceived pertussis vaccination in adults to be less important than did European physicians (61% of US physicians considered pertussis vaccination to be important, compared with 70% of French and German physicians combined; *p* = 0.015). Yet, physicians from each of the three countries placed the importance of vaccination against pertussis lower than four to five other vaccine-preventable adult diseases (Fig. 2).

To understand which subgroups of adult patients were perceived by physicians to be most at risk of pertussis, participating physicians were asked to select from a list of patient subtypes (Supplementary Fig. 1). Overall, those who are immunocompromised (559 [77%]), have COPD (522 [72%]), have other respiratory conditions (489 [67%]), or have asthma (475 [65%]) were considered to be at greatest risk of having severe consequences of pertussis.

Overall, the majority of surveyed physicians (608 [76%]) in all countries felt that the pertussis vaccination coverage rates in adults were suboptimal. Physicians also agreed that it is important to protect vulnerable persons against pertussis, either directly (603 [77%]) by vaccinating the vulnerable person or indirectly (619 [78%]) by vaccinating their close contacts.

### Section B

#### Physicians’ attitude and vaccination recommendation behavior

Physicians in all countries reported recommending Tdap vaccination for their adult patients according to the official recommendations; however, they also indicated that they were more likely to recommend Tdap vaccination to those in higher risk groups than in otherwise healthy adults. The rate at which physicians recommend Tdap vaccination for adults with COPD, asthma or other conditions – including diabetes, cardiovascular disease, obesity and others – was significantly higher than that for adults without medical issues, at 472 [59%], (439 [55%] and 400 [50%], respectively, vs. 327 [41%] (*p* < 0.005 for each pair of medical issue vs. no issue; Supplementary Fig. 2). The same pattern of behavior was observed across all three countries, but the recommendation rates were lower among French physicians. Overall, almost half of the physicians frequently recommended that their adult patients with asthma and/or COPD received a pertussis booster (Supplementary Fig. 3). This ratio was the highest among German physicians at 53% and lowest among French physicians at 32%.

### Patient vaccination journey in France

Traditionally, vaccination in medical offices in France requires that the patient take a prescription to the pharmacy, pick up the vaccine from the pharmacy, and then return to the medical office for vaccine administration. However, at the time of this study, the national pharmaceutical agreement that allows pharmacists to administer vaccines prescribed by physicians at the pharmacy had just came into effect [19] (pharmacists are now able to prescribe and administer certain vaccines at the pharmacy). The French physicians were asked to provide their perception on how this new agreement will affect the vaccination of their patients.

Only a quarter of the French physicians selected the response: ‘*I am glad that pharmacists may be able to assist with administering vaccines to increase the vaccination coverage rate and free up my practice for other patients*’. More than half of the physicians (110 [55%]) selected the response: ‘*I am concerned that I will not be able to check if the vaccine has been administered*’.

#### Physician-reported patient perception and behavior

Physicians reported that the public generally has a low awareness of adult vaccinations; only 159 (20%) physicians felt that adults’ awareness of the rationale for immunization was high. Overall, 209 (27%) physicians felt that patients were confident to request to be vaccinated proactively, while only 129 (17%) felt that patients proactively sought information on vaccines. When asked more specifically about pertussis vaccination, 323 (43%) physicians reported that pertussis vaccination was perceived positively by their patients.

When asked about their patients’ pertussis vaccination acceptance/uptake (based on their recall and not through formal assessment of medical records), physicians reported that approximately two in three patients who were recommended a pertussis vaccination received it (Supplementary Fig. 4). Overall, an estimated 70%, 69%, and 68%, respectively, of adults with COPD, asthma, or other conditions were perceived to have received a pertussis vaccination after it being recommended by their physician, compared with 65% of those without medical issues (*p* < 0.005, *p* = 0.013, and *p* = 0.018, respectively). Overall, of 709 physicians who reported patients not receiving pertussis vaccinations despite their recommendation, the reasons patients did not get vaccinated were given as: (i) low perception of personal risk – 432 (61%) physicians reported that their patients did not feel they were at risk for pertussis; (ii) attitudes against vaccination – 102 (14%) physicians reported that their patients did not believe in the need or usefulness of vaccination in general; (iii) low sense of urgency to be vaccinated and not keeping vaccination appointments – 244 (34%) physicians reported that their patients had prioritized other vaccine(s), and 105 (29%), 114 (67%), and 58 (33%) physicians from the US, France, and Germany, respectively, felt that their patients did not receive the vaccination due to those patients delaying or forgetting the vaccination appointment; (iv) efficacy/side effects – physicians reported that patients did not believe in the efficacy of the vaccine (170 [24%]) or feared the potential side effects (333 [47%]); and (v) cost – in total, 97 (27%) physicians from the US reported a belief that their patients did not receive the pertussis vaccination due to the vaccine being too expensive, compared with 3 (2%) and 8 (5%) physicians from France and Germany, respectively, where the vaccine cost is more easily covered (Table 2).

**Table 2:** Physician-reported reasons why patients did not receive pertussis vaccinations despite their recommendation

Physician-reported reasons, *n* (%)	Overall (*N* = 709)	US (*n* = 364)	France (*n* = 171)	Germany (*n* = 174)
Vaccine hesitancy in general	471 (66)	269 (74)	87 (51)	115 (66)
Patient does not feel at risk	432 (61)	238 (65)	92 (54)	102 (59)
Vaccine fatigue post-COVID-19	365 (51)	203 (56)	76 (44)	86 (49)
Fear of side-effects	333 (47)	183 (50)	62 (36)	88 (51)
Forget to be vaccinated after prescription was received	277 (39)	105 (29)	114 (67)	58 (33)
Prioritization of other vaccines(s)	244 (34)	154 (42)	43 (25)	47 (27)
Lack of belief in the efficacy of the vaccine	170 (24)	109 (30)	26 (15)	35 (20)
Time required to be vaccinated	118 (17)	60 (16)	29 (17)	29 (17)
Vaccine too expensive	108 (15)	97 (27)	3 (2)	8 (5)
Pertussis vaccination not required for patient	102 (14)	60 (16)	20 (12)	24 (14)

Response to the study question ‘Why did some of your patients not get vaccinated with Tdap/Tdap-IPV after your recommendation?’

Tdap, tetanus-diphtheria-acellular pertussis vaccine with reduced antigenic doses of diphtheria and acellular pertussis

#### Impact of COVID-19

The impact of the COVID-19 pandemic on physicians’ level of recommendation of pertussis vaccination to their adult patients appears to be limited – similar results were seen across the three countries. Among all surveyed physicians, 146 (18%) felt that the pandemic had increased or significantly increased their level of recommendation of pertussis booster vaccination in adults – 85 (21%) in the US, 31 (16%) in France, and 30 (15%) in Germany (Supplementary Fig. 5). While there was a small increase in physicians’ level of recommendation of pertussis boosters, the percentage of adult patients who received the vaccination, as perceived by physicians, was similar before and after the pandemic, at 67% (272 administrations out of 408 prescriptions) and 68% (296 administrations out of 432 prescriptions), respectively (*p* = 0.191).

### Section C

#### Information of interest

The types of information around pertussis and pertussis vaccination that most interested physicians varied across countries. US physicians are most interested in information on pertussis vaccine efficacy and patient populations that are most at risk of pertussis; French physicians are most interested in information on patient populations that are most at risk of pertussis and automatic reminders of vaccination schedules; German physicians are most interested in information on pertussis-vaccine efficacy and vaccine co-administration guidance (Supplementary Fig. 6).

#### Preferred tools and channels

For educational activities that allow physicians to learn more about adult patients most at risk for pertussis, 304 (38%) physicians overall reported preferring Continuing Medical Education (CME) programs or public educational campaigns, and 216 (27%) reported that improved screening materials (such as screening tests or data relating to risks in specific patient groups) would be helpful in decision-making.

Digital tools, including electronic medical health records, automatic reminders, or electronic tracking applications were reported by 200 (25%) physicians to help in performing their daily tasks and assessing patients’ vaccination histories and statuses.

## Discussion

This study was designed to assess physicians’ perceptions of pertussis vaccination in non-pregnant adults, focusing on its importance for individual disease prevention in three countries that have routine recommendations for Tdap vaccination in adults. Our results show that while most physicians felt that pertussis vaccination in adults is important, this concern was often not reflected in their vaccination practices.

Across the three countries, there were broadly similar findings regarding physicians’ perception of which groups are at highest risk and pertussis vaccination recommendation behaviors.

### Physicians’ attitudes and vaccination recommendation behavior

#### In healthy adults

In this study, the majority of physicians agreed that it is important to protect people who are most at risk of pertussis but also noted that the vaccination coverage rates in adults were at a suboptimal level in their respective countries. Indeed, data suggest coverage of pertussis vaccination in adults was low, at only 30.1% in 2019 in the US [20], 38.5% in 2018 in France (for those aged 29 years; coverage was below 27% for those aged 49 years and over) [21], and 49.8% in 2016–2021 in Germany [22]. These percentages are concerning, especially as data have shown that adults are now proportionally more affected by pertussis compared with 10–20 years earlier [23].

The perception of pertussis being a childhood disease and the underestimation of the disease burden in adults can be seen in physicians’ perception of the relative unimportance of pertussis vaccination as compared with other adult vaccines. There is a lack of awareness of how pertussis can affect even healthy adults. Medical societies and recommending bodies should enhance efforts to protect adults against pertussis through vaccination, aiming to reduce morbidity and mortality in this population and decrease the circulation of the infectious agent.

#### In high-risk adults

While pertussis may be under-recognized in the broad adult population, surveyed physicians appeared to be more aware of the potential severe complication of pertussis in those with chronic medical conditions, such as COPD and/or asthma, as seen in the increased level of pertussis vaccination recommendation in patients with these conditions compared with those without medical issues. Yet, while the level of vaccine recommendation was higher for these vulnerable adults, overall, this level was moderate, at under 60%. This underscores the need for recommending bodies in the three countries to highlight the increased vulnerability of individuals with high-risk conditions and emphasize the importance of ensuring these patients are vaccinated against pertussis to prevent severe complications.

Across all three countries, physicians regarded immunocompromised patients as the adult population most at risk of pertussis, followed by patients with COPD. In our study, the frequency at which physicians recommend adults with asthma and/or COPD be vaccinated against pertussis was modest. These data show that even high-risk patients are not being prioritized for protection against pertussis.

Would national guidelines for vaccinating high-risk adults improve vaccination rates in this cohort? It is unclear whether a risk-based approach would effectively increase vaccination rates among these adults. Developing specific recommendations for adults with conditions that put them at risk for severe pertussis could increase focus on this group, similar to recommendations for those at risk of influenza or pneumococcal disease. However, this approach might detract from promoting routine pertussis booster vaccination throughout the lifespan. Additionally, there is no guarantee that targeted vaccination recommendations would lead to higher vaccine uptake, as evidenced by the low pneumococcal vaccination coverage in high-risk patients in France despite specific recommendations [24, 25]. Regardless of the approach, healthcare professionals play a vital role in advocating for the value of vaccination to protect their patients’ health.

### Physician-reported patient perception and behavior

In all three countries, physicians felt that awareness of vaccination among their adult patients was low, with only 20% believing it to be high. They also reported that few adult patients proactively ask to be vaccinated. This indicates that many adults rely heavily on their doctors for information, guidance, and recommendations [26, 27]. Patients’ trust in their doctor’s competence and care significantly impacts patient outcomes [28]. Therefore, physicians play a critical role in increasing awareness, educating patients on the risk of pertussis, identifying those most at risk of severe disease, promoting vaccination, and alleviating any concerns about vaccination.

Patients’ adherence to physicians’ vaccination recommendations was reported to be moderate. Based on physicians’ recall (not through formal assessment of medical records), when a recommendation for pertussis vaccination was given to their adult patients without medical issues, approximately two in three patients were vaccinated. When looking at vaccine uptake among adults with asthma and/or COPD vs. healthy adults, conversion rates (from vaccine recommendation to receipt of vaccination) were perceived to be similar by French and German physicians. The similar perceived conversion rates between adults with medical issues vs. those without in France and Germany could again be attributed to the lack of national recommendations in pertussis vaccination in these high-risk groups – high-risk patients may not be advised of their increased risk of severe consequences of pertussis.

### Impact of COVID-19

COVID-19 has posed substantial challenges to public health systems worldwide. While the pandemic increased public awareness of the benefits of vaccination programs and acceptance of other vaccines [29, 30], our study found that the percentage of adult patients receiving pertussis boosters remained similar before and after the pandemic. Physician-reported data suggest that the COVID-19 pandemic had a limited impact on their behavior regarding pertussis vaccine recommendations for adults, and there was no notable increase in patient acceptance of these recommendations when they were given. This may be because their recommendations are primarily guided by national guidelines and policies, making them less susceptible to external influences. Additionally, the overall low adult pertussis vaccination rates in these countries could also be a factor. The reasons behind these findings remain unclear.

### Facilitators and barriers to pertussis vaccination recommendations

The suboptimal implementation of existing recommendations on vaccination against pertussis for the broad adult population among physicians in the US, France, and Germany is a concern. Further, it is apparent from this study that not all physicians are aware of the potential for increased risk of pertussis infection among those with respiratory conditions such as asthma and/or COPD. This once again emphasizes the need to raise physicians’ awareness of pertussis in these high-risk patients in order to better prioritize their recommendations.

Traditionally, vaccination in France was a time-consuming task for both individuals and physicians in that vaccines weren’t stocked in the physician’s office. A patient had to get a vaccine prescription from the doctor, visit the pharmacist to pick up the vaccine and then make another appointment with their doctor for the vaccine to be administered. This inconvenience may have contributed to the low uptake of pertussis boosters in the country, as individuals may forget either to pick up the vaccine from the pharmacist or to make a second appointment to see their doctor for its administration. These impediments are reflected in physicians’ responses, in which low vaccine uptake in patients due to their delaying or forgetting the appointment was the highest in France (67%) compared with the US (29%) and Germany (33%); the latter two countries provide vaccination in their physician’s office or at a pharmacy [31]. The French national pharmaceutical agreement that allows pharmacists to administer certain vaccines could drastically increase vaccine coverage in France in the coming years. A quarter of the surveyed French physicians held a positive attitude towards this change, but 55% were concerned as they may have no visibility as to whether the prescribed vaccine was indeed taken up by the patient.

The public relies heavily upon physicians’ knowledge of vaccination in general and on specific vaccines, so continued updating is critical. Information on vaccine efficacy and safety, patient groups who are most at risk of pertussis and guidance on vaccine co-administration were the topics reported by physicians to help them with their pertussis vaccination recommendations.

### Strengths and limitations

The strengths of this study were the survey design, including a broad scope of questions with steps taken to ensure that questions minimized any potential bias. However, limitations of this study are that selection bias cannot be excluded, as participating physicians were selected solely from one database, which could indicate a homogenous population of responders. However, the selection of physicians from several countries was felt to help offset such bias. Further, the number of participating physicians was not equally distributed between the three countries; while results were largely similar between countries, overall responses were weighted towards those from the US. Payment for participation may have created a reporting bias, and differences between respondents in the interpretation of survey questions may also be possible. Finally, it should be noted that the responses, particularly regarding vaccine recommendations given and accepted, are the physicians’ own perceptions and not from official databases.

## Conclusions

While pertussis vaccination in adults is included in national recommendations, implementation is regrettably infrequent and inconsistent. Our behavioral study highlights that the importance of vaccination against pertussis is ranked lower than that of COVID-19, influenza, pneumococcal disease, and tetanus. It would be beneficial to increase physicians’ awareness of the value of pertussis vaccination in vulnerable adults who are at risk of severe complications, including those with asthma and/or COPD, but improved awareness may have limited effect unless vaccination of vulnerable groups is included in official recommendations. In addition, pertussis vaccine uptake is suboptimal even when a vaccination has been recommended. Digital reminders that a patient’s vaccinations are due and access to patients’ vaccination status were reported by physicians to be the most helpful tools to aid in their level of recommendation of pertussis vaccination in adults. Such efforts could lead to improved vaccine coverage, and a reduced risk of this vaccine-preventable disease.

## Electronic supplementary material

Below is the link to the electronic supplementary material.

Supplementary Material 1

## Data Availability

The datasets used and/or analysed during the current study are available from the corresponding author on reasonable request.

## Abbreviations

CDC: Centers for Disease Control and Prevention
CME: Continuing Medical Education
COPD: chronic obstructive pulmonary disease
EphMRA: European Pharmaceutical Market Research Association
GDPR: General Data Protection Regulation
IPV: inactivated poliovirus
Tdap: tetanus-diphtheria-acellular pertussis vaccine with reduced antigenic doses of diphtheria and acellular pertussis
