# Supplementary material for: Pertussis vaccination in adults: a behavioral study of physicians from the US, France, and Germany
Source: BMC Prim Care. 2024 Nov 26;25:402. doi: 10.1186/s12875-024-02647-3 (PMC11590461; doi:10.1186/s12875-024-02647-3)
Supplement: Supplementary file 1 — Supplementary Material 1 [file 12875_2024_2647_MOESM1_ESM.docx]

**Pertussis vaccination in adults: a behavioral study of physicians from the US, France, and Germany – supplementary material**

# Methods

## Physician eligibility criteria

Physicians must:

1. be personally involved in the decision to vaccinate adults and prescribe Tdap (or Tdap-IPV) vaccination
2. be in practice for more than 3 years and less than 44 years
3. see a majority of adult patients, including a good proportion of those with asthma and/or COPD
4. vaccinate their patients with Tdap/Tdap-IPV
5. see more than 100 adult patients per month (France), more than 70 per month (US) or more than 150 per quarter (Germany)
6. have at least 50% adult patients (18 years old and above) in their caseload
7. see a minimum of 10 adult patients with COPD or asthma per month
8. prescribe a minimum of 10 doses of Tdap (or Tdap-IPV) vaccination per month (US), or 5 doses per month (France and Germany)
9. have a self-reported board certification (US only).

## Physician questionnaire

**Section A – Your awareness and knowledge of pertussis**

A1. What is your perception of the need to vaccinate against the following diseases when thinking of your **adult patients**? Please rank from very low need (1) to very high need (7).

**RANDOMIZE**

1. Pertussis (whooping cough)
2. Hepatitis A
3. Hepatitis B
4. Human papillomavirus (HPV)
5. Meningococcal disease
6. Diphtheria
7. Influenza
8. COVID-19
9. Poliomyelitis
10. Pneumococcal disease
11. Tetanus
12. Herpes zoster

A2. Thinking of **pertussis**, which words come to mind immediately?

A3a. How concerned are you about pertussis in **your adult patients**, if at all? Please select from 1 (I am not concerned at all about it) to 7 (I am extremely concerned about it).

A3b. How often do you see adults with pertussis in your practice?

1. Never
2. Rarely
3. Occasionally/sometimes
4. Often/frequently

A3c. To the best of your knowledge, which of the following groups of adult patients are **most at risk of pertussis**?

**MULTICODE. RANDOMISE**

1. Patients with asthma
2. Patients with COPD
3. Smokers
4. Obese patients
5. Immunocompromised patients
6. Patients with neurologic disorders
7. Pregnant women
8. Patients facing occupational hazards (e.g. care role working with children, those working with animals, those working in hospital settings)
9. Elderly patients (65+ years old)
10. Adults who are in contact with young infants
11. Patients with other respiratory conditions (excluding asthma and/or COPD)
12. Patients with diabetes
13. Patients with cardiovascular disease
14. Other, please specify
15. None of the above
16. I don’t know

A4. What type of services or tools do you think could help identify the groups of adult patients most at risk of pertussis to ensure vaccination?

**Section B – The current pertussis-containing booster vaccines and impact of COVID-19**

B1a. To what extent (if any) would you say the **COVID-19 pandemic** has impacted on your [**NOT US:** prescribing] [**US ONLY:** recommendation] of **pertussis**-containing booster vaccines? Please rate from 1 (significantly decreased) to 7 (significantly increased).

B1b. Regardless of current national/local recommendations, approximately to what proportion of adult patients (18+ years old) do you [**FR:** prescribe/**DE:** prescribe/recommend/**US:** recommend] pertussis-containing booster vaccines in a typical [**FR**/**US:** month/**DE:** quarter]?

*Please note that recent data shows that adults with asthma and/or COPD may have more severe consequences of pertussis infection/disease.*

**OPEN NUMERIC QUESTION; RANGE 0–100 FOR EACH CELL**

| **Patient group** | % of adult patients to whom I [**NOT US** prescribed/**US ONLY** recommended] **pertussis**-containing booster vaccines **PRIOR to the COVID‑19 pandemic** | % of adult patients to whom I [**NOT US** prescribed/**US ONLY** recommended] **pertussis**-containing booster vaccines **POST-COVID-19 pandemic** |
| --- | --- | --- |
| 1. Adults without medical issues | % | % |
| 1. Adults with **asthma** | % | % |
| 1. Adults with **COPD** | % | % |
| 1. Adults with **any other medical conditions** (e.g. diabetes, cardiovascular disease, obesity, etc.) | % | % |

B2. Out of your adult patients to whom you have [**FR:** prescribed/**DE:** prescribed/recommended/**US ONLY: recommended**] a **pertussis-containing** booster vaccine, what proportion **received it**? Please provide the percentage of adult patients receiving pertussis-containing booster vaccines prior to and post-COVID-19 pandemic.

1. Adults without medical issues to whom I have [**NOT US** prescribed/**US ONLY** recommended] a pertussis-containing booster vaccine.
2. Adult patients with **asthma** to whom I have [**NOT US** prescribed/**US ONLY** recommended] a pertussis-containing booster vaccine.
3. Adult patients with **COPD** to whom I have [**NOT US** prescribed/**US ONLY** recommended] a pertussis-containing booster vaccine.
4. Adults with any other medical conditions (e.g. diabetes, cardiovascular disease, obesity, etc.) to whom I have [**NOT US** prescribed/**US ONLY** recommended] a pertussis-containing booster vaccine.

B3a. Why did some of your patients not get vaccinated with [**DE&US:** Tdap (tetanus, diphtheria, pertussis)/**FR:** dTcaP & **DE:** Tdap-IPV (tetanus, diphtheria, pertussis, poliomyelitis)] after your recommendation?

B3b. Why did some of your patients not get vaccinated with [**DE/US:** Tdap/**DE/FR:** Tdap-IPV/**FR:** dTcaP] after your recommendation?

*Please select all that apply, even if you have already mentioned it in the previous question.*

**MULTICODE. RANDOMIZE**

1. It is too expensive for some patients.
2. Some patients forget to do the vaccination once they received the prescription.
3. Some patients prioritize other vaccine(s).
4. Some patients do not feel at risk.
5. Some patients are vaccine hesitant in general.
6. Some patients have become vaccine hesitant post-COVID-19.
7. The pertussis vaccination is not required for these patients.
8. Patient fear of side effects.
9. Time required to get vaccinated.
10. Some patients do not believe in the efficacy of the Tdap vaccine.
11. Other, please specify.

B4. Thinking about the discussions you have had with your patients about the pertussis-containing booster vaccines in the past, for what proportion:

|  | **Adults without medical issues**  a. | Adult patients with **asthma**  b. | Adult patients with **COPD**  c. | Adults with **any other comorbidities** (diabetes, cardiovascular disease, obesity, etc.)  d. |
| --- | --- | --- | --- | --- |
| 1 …did **you initiate** the conversation? | % | % | % | % |
| 2 …did **the patient initiate** the conversation? | % | % | % | % |
| 3. Don’t know | % | % | % | % |
| TOTAL | **MUST SUM TO 100%** | **MUST SUM TO 100%** | **MUST SUM TO 100%** | **MUST SUM TO 100%** |

B5. To what extent do you agree with the following statements? Please select from 1 (strongly disagree) to 7 (strongly agree).

**RANDOMIZE**

- 1. It is important to [**FR:** prescribe/**DE:** prescribe/recommend/**US:** recommend/administer] pertussis-containing booster vaccines **to avoid disease resurgence.**
  2. It is important to [**FR:** prescribe/**DE:** prescribe/recommend/**US:** recommend/administer] pertussis-containing booster vaccines **to directly protect vulnerable adults** (**e.g.** those at higher risk of infection or severe disease).
  3. It is important to [**FR:** prescribe/**DE:** prescribe/recommend/**US:** recommend/administer] pertussis-containing booster vaccines to my patients **to ensure indirect protection of vulnerable persons in contact with them/in their surroundings** (newborns, patients with chronic illnesses, immunocompromised…).
  4. I believe in the **value** of pertussis-containing booster vaccines **in adults at risk of pertussis** (i.e. those with a respiratory condition) for individual protection.
  5. It is **important to** protect certain patients most at risk of pertussis (i.e. those with a respiratory condition) against pertussis.
  6. My **patients proactively seek information** on vaccines (incl. pertussis-containing booster vaccines).
  7. My **patients feel** confident to actively ask to be vaccinated.
  8. The overall awareness of the lay public about immunization is high.
  9. The vaccination coverage rates of pertussis-containing booster vaccines among adults at risk of pertussis (i.e. those with a respiratory condition) are at an optimal level.
  10. Pertussis-containing booster vaccines are perceived positively by my patients.

B6. We want to understand now to what extent the COVID-19 pandemic changed your/your patients’ behavior regarding vaccination overall. To what extent do you agree with the following statements? Please select from 1 (strongly disagree) to 7 (strongly agree).

**RANDOMIZE**

1. The COVID-19 pandemic has increased my awareness of what defines a **vulnerable adult** (e.g. those at higher risk of infection or severe disease).
2. Since the COVID-19 pandemic, I see increased **value in immunization of vulnerable adults** (e.g. those at higher risk of infection or severe disease).
3. Since the COVID-19 pandemic, I see increased **value** in **pertussis-containing** booster vaccines in adults at risk of pertussis (i.e. those with a respiratory condition) for **individual protection**.
4. Since the COVID-19 pandemic, there is a greater **need to** protect the patients most at risk of pertussis (i.e. those with a respiratory condition).
5. Since the COVID-19 pandemic, more **patients seek information** on vaccines (incl. pertussis-containing booster vaccines).
6. Since the COVID-19 pandemic, **the overall awareness** of the **lay public** about immunization has increased.
7. Since the COVID-19 pandemic, **lay public willingness** to get vaccinated to protect themselves has increased.
8. Since the COVID-19 pandemic, **lay public willingness** to get vaccinated to protect others has increased.
9. Since the COVID-19 pandemic, the **lay public perceives the value of booster** **vaccination** in adults for individual protection.
10. Since the COVID-19 pandemic, the vaccination coverage rate of pertussis-containing booster vaccines among adults at risk of pertussis (i.e. those with a respiratory condition) has decreased.
11. It is a priority to catch up on the pertussis-containing booster vaccinations missed due to the COVID-19 pandemic.

B7. Please select the statement that best describes your current knowledge of the vaccination schedule for pertussis-containing booster vaccines for the adult population in your country. 1 = I am not knowledgeable at all about it; 4 = I am moderately knowledgeable about it; 7 = I am extremely knowledgeable about it.

B8. Which sources do you use primarily as a basis for your vaccine recommendations to your adult patients?

**MULTIPLE CHOICE. RANDOMIZE**

1. National guidelines/national immunization program/**[FR]** Calendrier des vaccinations
2. Medical society guidelines
3. **[DE]** The STIKO
4. **[FR]** HAS (Haute Autorité de Santé)
5. I use my experience in assessing patients at risk of pertussis who are good candidates
6. **[US]** ACIP/CDC (Advisory Committee on Immunization Practices/The Centers for Disease Control and Prevention)
7. **[US]** AAFP recommendations (American Academy of Family Physicians)
8. **[US]** ACP recommendations (American College of Physicians)
9. **[US]** State department of health
10. Others (please specify)

B9. In the next question we will ask you for your level of agreement regarding messages on pertussis. To what extent do you agree with the following statements? Please select from a (strongly disagree) to g (strongly agree).

**RANDOMIZE**

1. Pertussis is a highly contagious respiratory disease that also affects adults and may lead to complications.
2. There is no lifelong protection against pertussis after either natural infection or childhood immunization, so there is a need for pertussis booster vaccination throughout life.
3. [**DE& US:** Tdap/**DE:** Tdap-IPV/**FR:** dtcaP] booster vaccination for adults ensures continuity of protection (direct and indirect) throughout their lifetime – especially important for adults with underlying medical conditions.
4. [**DE& US:** Tdap/**DE:** Tdap-IPV/**FR:** dTP/dtcaP] booster programs should be a cornerstone of established adult immunization schedules worldwide.
5. Pertussis-containing booster vaccines [**US:** need to be/**FR, DE:** should be] administered in adults every 10 years and during each pregnancy.

B10. Which procedures do you integrate into your daily practice in order to be able to vaccinate your patients against pertussis? *Please select yes or no for each.*

**RANDOMIZE**

1. When patients come into my practice for the first time, I check their vaccination status on a routine basis.
2. When patients come into my practice for the first time, I check their **pertussis-containing booster vaccination status** on a routine basis.
3. I use a reminder system for vaccinations (including **pertussis-containing** booster vaccines), i.e. software.
4. My practice reminds my patients actively about overdue vaccines, i.e. via phone call, SMS or letter.
5. I display information material in the waiting area in order to inform my patients about the risks of pertussis (whooping cough) and prevention through vaccination.
6. When patients come for another adult vaccination, I also offer to concomitantly administer a **pertussis-containing** booster vaccine if eligible.

B11a. You have mentioned that you routinely check your patients’ pertussis booster vaccination status. How do you do this?

**MULTIPLE CHOICE. RANDOMIZE**

1. I check my patient’s vaccination record.
2. I ask my patient directly.
3. [**FR:** dans le dossier médical partagé].
4. Patient recall systems for automatic notifications.
5. Other, please specify.

B11b. With which other vaccine(s) do you co-administer pertussis-containing booster vaccines?

**MULTIPLE CHOICE. RANDOMIZE**

1. COVID-19 vaccine
2. Seasonal influenza vaccine
3. Pneumococcal vaccine
4. Herpes zoster vaccine
5. Human papillomavirus vaccine
6. Meningitis vaccine
7. Other, please specify

B12. How frequently do you check **pertussis-containing** booster vaccination status for the following adult patient groups?

|  | Never | Less than yearly | Yearly | Every 6 months | Quarterly | Monthly | Always, during every visit |
| --- | --- | --- | --- | --- | --- | --- | --- |
| 1. Adults without medical issues |  |  |  |  |  |  |  |
| 1. Adult patients with **asthma** |  |  |  |  |  |  |  |
| 1. Adult patients with **COPD** |  |  |  |  |  |  |  |
| 1. Adults with **any other conditions** (diabetes, cardiovascular disease, obesity, etc.) |  |  |  |  |  |  |  |

B13. When the status of the **pertussis** vaccination is **unknown**, what is your typical behavior for the following adult patient groups?

|  | I [**FR:** prescribe/ **DE:** prescribe/ recommend/ **US:** recommend/ administer] **pertussis-containing** booster vaccine | **I do not** [**FR:**prescribe/ **DE:** prescribe/ recommend/ **US:** recommend/ administer] **pertussis-containing** booster vaccine | **Don’t know** |
| --- | --- | --- | --- |
| 1. Adults without medical issues |  |  |  |
| 1. Adult patients with **asthma** |  |  |  |
| 1. Adult patients with **COPD** |  |  |  |
| 1. Adults **with any other conditions** (diabetes, cardiovascular disease, obesity, etc.) |  |  |  |

B14. Do you currently [**FR:** prescribe/**DE:** prescribe/recommend/**US:** recommend/administer] **pertussis-containing** booster vaccines to your **adult patients with asthma and/or COPD**? Please select your answer from 1 (never) to 7 (all the time).

B15. Why are you currently not [**NOT US:** recommending/prescribing/administering/**US:** recommending/administering] **pertussis-containing** booster vaccines to your **adult patients with asthma and/or COPD**?

**MULTICODE. RANDOMIZE WITHIN EACH GROUP. RANDOMIZE ORDER OF GROUPS SHOWN**

**Reasons related to me:**

1. It is **not a priority for me** to vaccinate against pertussis compared to other diseases.
2. **I don’t know which adults** should receive pertussis-containing booster vaccines.
3. **I** **always [NOT US:** **prescribe/US: administer] the same booster vaccine brand to all my adult patients.**
4. **I** **am not the one responsible** for pertussis vaccination for at-risk patients; another HCP (e.g. pulmonologist, pharmacists, PCP, nurse) is.
5. **No time to discuss** with all patients during a visit.
6. I am **concerned about the efficacy** of **pertussis-containing** booster vaccines in adults.
7. I am **concerned about the safety** of **pertussis-containing** booster vaccines in adults.
8. I am **not convinced** that adults require a **pertussis-containing** booster vaccine as pertussis is not severe for this population.

**Reasons related to patients:**

1. It is **not a priority for the adult patient** to get vaccinated against pertussis.
2. **Patients do not proactively request** pertussis vaccination.
3. **Patient lack of knowledge about the diseases** being prevented.
4. **Patient lack of awareness/knowledge about the vaccine.**
5. **Patient concerns about adverse events**/side effects.
6. **Patient concerns about the lack of efficacy** of the vaccine.
7. Pertussis is perceived as a **childhood disease** **by patients** and therefore they do not feel concerned.
8. Patients **drop off between prescription and vaccination.**

**Other reasons**

1. There is a **lack of clear understanding on who** (which healthcare professional) should recommend the vaccine.
2. There is a **lack of clear understanding on when** (at which age) the vaccine should be recommended based on official guidelines.
3. There is a **lack of clear recommendation or information on which patients** **are at risk** for pertussis.
4. There is **no regular wellness checkup** or other opportunity to [**NOT US:** prescribe/**US:** recommend]/check vaccination status.
5. The **vaccination status is not always known**/tracked.
6. **No effective reminder system** in place for healthcare professionals.
7. The **long-term complications** of pertussis are unknown.
8. **Vaccine shortage.**
9. No availability of monovalent pertussis vaccine.
10. Other, please specify.

B16. What could help you improve your [**FR:** prescribing/administering/**DE:** recommending/prescribing/administering/**US:** recommending/administering] **of pertussis-containing** booster vaccines to your **adult patients at risk of pertussis** (e.g. suffering from asthma and/or COPD)? *Please be as descriptive and detailed as possible, your answer is important to us.*

B17. To what extent are you aware of the following? Please select from 1 (not at all aware) to 7 (extremely aware).

**SINGLE CODE PER ROW/ RANDOMISE**

1. **Adults with asthm**a are up to 4x more likely to get pertussis.
2. **Adults with asthma** are 40% more likely to be hospitalized for pertussis than patients without asthma.
3. **Adults with COPD** are up to 3.5x more likely to get pertussis.
4. **Adults with COPD** are 75% more likely to be hospitalized for pertussis than patients without COPD.

B18. How do the following statements impact your motivation to [**NOT US**: recommend/prescribe]/ [**US**: recommend/administer] pertussis-containing booster vaccine, if at all? Please select from 1 (strongly discourage me) to 7 (strongly encourage me).

Impact on my motivation to [**FR**: prescribe/ **DE**: prescribe/recommend/[**US**: recommend/administer] pertussis-containing booster vaccine in **adults (overall)**.

1. Pertussis is up to 10x more contagious than influenza.

Impact on my motivation to [**FR**: prescribe/ **DE**: prescribe/recommend/[**US**: recommend/administer] pertussis-containing booster vaccine in **adults with asthma**.

1. **Adults with asthma** are up to 4x more likely to get pertussis.
2. **Adults with asthma** are 40% more likely to be hospitalized for pertussis.

Impact on my motivation to [**FR**: prescribe/ **DE**: prescribe/recommend/[**US**: recommend/administer] pertussis-containing booster vaccine in **adults with COPD**.

1. **Adults with COPD** are up to 3.5x more likely to get pertussis.
2. **Adults with COPD** are 75% more likely to be hospitalized for pertussis.

**Section C – The future of pertussis-containing booster vaccine recommendations**

We would like to focus on **pertussis-containing** booster vaccines for **adult patients (18+ years old).**

C1. Please indicate the extent to which you expect your future [**FR:** prescription/**DE:** prescription/recommendation/**US:** recommendation/administration] of **pertussis-containing** booster vaccines to change for each of the following adult patient groups. Please select from 1 (significantly decrease) to 7 (significantly increase).

1. Adults without medical issues
2. Adult patients with asthma
3. Adult patients with COPD
4. Adults with any other conditions (diabetes, cardiovascular disease, obesity, etc.)

C2a. Why would you expect your future [**FR:** prescription/**DE:** recommendation/prescription/**US:** recommendation] of **pertussis-containing** booster vaccines to **decrease** for the patient groups below?

Please select all that apply.

1. Pertussis is not a priority compared to other vaccinations.
2. Pharmacists are becoming more and more involved in adults’ vaccination, so I am less involved in vaccination recommendation for adults.
3. Fewer patients’ visits in doctors’ office since COVID-19.
4. Vaccination fatigue due to COVID-19.
5. Other, please specify.

C2b. Why would you expect your future [**FR:** prescription/**DE:** recommendation/prescription/**US:** recommendation/administration] of **pertussis-containing** booster vaccines to **increase** for the patient groups below?

Please select all that apply.

1. Catch up on booster vaccinations missed during COVID-19
2. Increased awareness of importance of vaccination, esp. with respiratory conditions, since COVID-19 among HCPs
3. Increased awareness of importance of vaccination among patients
4. I am now aware that adults with asthma and/or COPD are at increased risk if they get pertussis
5. I am now aware that adults with asthma and/or COPD are more likely to be hospitalized for pertussis
6. With pharmacists now vaccinating, patients would have more opportunities for vaccination and thus ask more for vaccination
7. New guidelines for specific risk groups from national authorities or societies
8. Outbreaks of pertussis in some communities
9. Proactive recommendation from their doctor and/or pharmacist
10. Other, please specify

C3a. Please select and rank the top 5 services/changes that would motivate you to [**FR:** prescribe/**DE:** prescribe/recommend/**US:** recommend] more **pertussis-containing** booster vaccines in **adult patients (18 years old +) in the future**. *1 = most important, 2 = second most important, 3 = third most important reason and so on. Please note no ties allowed.*

**MULTICODE. RANDOMIZE**

1. WHO (World Health Organization) recommendation
2. NITAG (National Immunization Technical Advisory Groups) recommendation **[NOT DE]**
3. Recommendations from national scientific societies for my professional bodies
4. Recommendation from the international scientific societies for my professional body
5. Access to vaccination status
6. Notification pop-up on the vaccination status
7. Reminders to you
8. Reminders to the patient
9. Medical training webinars
10. CME (continuing medical education) modules
11. Discussion guide to facilitate the discussion with patients
12. Cheat sheet to facilitate the discussion with patients
13. Proof of value (POV) material leaflet
14. Poster banners
15. Financial incentive for pertussis-containing booster vaccines in adults realigned with COVID‑19 and influenza
16. Vaccination coverage rate-based financial incentive
17. Co-administration guidance on adults’ vaccines
18. Epidemiologic data and/or data on long-term complications of pertussis
19. Pharmaceutical company information on vaccination recommendations (through reps)
20. **[US ONLY]** Insurance coverage for 65+
21. Other, please specify

C3b. Thinking of your adult patients **aged 65-year-old +**, what motivates you to [**FR:** prescribe/**DE:** prescribe/recommend/**US:** recommend] **Tdap-IPV** (tetanus-diphtheria-**pertussis**-polio) **instead of Td-IPV (tetanus-diphtheria-polio) only**?

**MULTICODE. RANDOMIZE**

1. The need to protect adults against pertussis
2. To simplify my practice, I use only Tdap-IPV
3. Due to the lack of clarity of the recommendations, hence defaulting to pertussis
4. I am not sure about the brand that contains pertussis or not
5. Cocooning for newborns
6. Other, please specify
7. None of the above

C4. What type of information are you most interested in finding out about **pertussis** disease and pertussis-containing booster vaccines for adult patients (18+ years old)? Please select all that apply.

**MULTICODE. RANDOMIZE**

1. Vaccine efficacy
2. Information related to the disease in adults
3. Available brands
4. Safety information for HCPs
5. Safety information for patients
6. Epidemiology data
7. Reminder of vaccination schedule (intervals, delays, catching up for foreigners/overseas students)
8. Data on long-term complications of pertussis disease
9. Co-administration guidance
10. Patients most at risk
11. Exceptional cases (e.g. pregnancies, allergies, patients on chemotherapy, patients with autoimmune diseases)
12. Information on local context and local population (e.g. incidence rate of disease)
13. Information on vaccinating in specific patient groups (e.g. age groups, at-risk patients)
14. Discussion guide to facilitate the discussion with patients
15. Cheat sheet to facilitate the discussion with patients
16. Other, please specify

C5. How do you prefer to receive information about **pertussis-containing** booster vaccines for adult patients (18+ years old)? *Please select and rank your 3 most preferred options.*

**MULTICODE**

1. In-person visits from a sales rep
2. Emails from pharmaceutical companies
3. Telephone calls from a sales rep
4. Letters from pharmaceutical companies
5. Pharmaceutical companies’ websites
6. In-person conference exhibits, presentations
7. Virtual conference exhibits, presentations
8. Online webinars
9. Journals/publications (online and paper)
10. Social media (Twitter, LinkedIn, Facebook, others)
11. Podcasts
12. Medical education events provided by pharmaceutical companies (virtual and in-person)
13. Flyers
14. Government-approved websites
15. Physician association resources
16. Colleagues and peers
17. Medical app(s) [**US:** e.g. UpToDate]
18. Other, please specify

C6. To what extent do you expect your use of the following digital channels to change over the next year to find information about **pertussis-containing** booster vaccines for adult patients (+18 years old)? Please select from 1 (significantly decrease) to 7 (significantly increase), or “I never use this channel and I don’t plan to use it over the next year”.

1. Emails from vaccine/pharmaceutical companies
2. Vaccine/pharmaceutical companies’ websites
3. Virtual conference exhibits, presentations
4. Online webinars
5. Online journals/publications
6. Social media (Twitter, LinkedIn, Facebook, others)
7. Podcasts
8. Virtual medical education events provided by vaccine/pharmaceutical companies
9. Government-approved websites
10. Physician association resources (online)
11. Medical app(s) [**US:** e.g. UpToDate]

C7a. Now that pharmacists are able to administer vaccines to adult patients on site **with** a prescription from a physician, which of the following statements best reflects your point of view?

**MULTIPLE CODE**

1. I am concerned that the pharmacists may take some of my patients away from my practice.
2. I am concerned that I will not be able to check if the vaccine has been administered.
3. I am glad that pharmacists may be able to assist with administering vaccines to increase the vaccination coverage rate and free up my practice for other patients. **EXCLUSIVE**
4. I do not know what to think about this. **EXCLUSIVE**

C7b. In the future, pharmacists may be able to administer vaccines to adult patients on site **without** a prescription from a physician. Which of the following statements best reflects your point of view?

**MULTIPLE CODE**

1. I am concerned that the pharmacists may take some of my patients away and impact my practice.
2. I am concerned that the pharmacists may take some of my patients away from my practice and that I will not be able to check if the vaccine has been administered.
3. I am glad that pharmacists may be able to increase the vaccination coverage rate and free up my practice for other patients. **EXCLUSIVE**
4. I do not know what to think about this. **EXCLUSIVE**

C7c. In the future, pharmacists’ role could change and they may be able to administer vaccines. Which of the following statements best reflects your point of view on this topic?

1. Pharmacists should **be allowed** to administer vaccines to adult patients.
2. Pharmacists should **not** be allowed to administer vaccines to adult patients.
3. I do not know.

C7d. **[FR & DE ONLY]** Considering that pharmacy-based immunization in adults is allowed and implemented, what impact would this have on the behavior you would adopt?

|  | 1. Stop | 1. Continue but decrease | 1. Stay the same | 1. Increase |
| --- | --- | --- | --- | --- |
| 1. My recommendation and [**NOT US** prescription] of pertussis-containing booster vaccines in adult patients will… |  |  |  |  |
| 1. My checks of the booster vaccination status for my adult patients will… |  |  |  |  |

# Supplementary tables and figures

**Table 1** Local recommendation of Td/Tdap for adults in the US, France, and Germany

|  | **US [1–4]** | **France [5–7]** | **Germany [8–10]** |
| --- | --- | --- | --- |
| **Recommended vaccine types** | Tdap and Td | Td-IPV and Tdap-IPV | Td, Tdap, and Tdap-IPV |
| **Adults** | Adults who have never received Tdap should receive a dose of Tdap, followed by a booster dose of either Tdap or Td every 10 years, or after 5 years in the case of a severe or dirty wound or burn | Boosters are recommended for adults every 20 years at the ages of 25 (Tdap-IPV), 45, and 65 years (mainly Td-IPV^a^), and every 10 years after the age of 65 years^a^ | All adults should receive a dose of Tdap^b^ once, followed by Td boosters every 10 years. Adults in risk groups (as detailed below) should receive Tdap^b^ every 10 years |
| **Pregnant women and household members (cocooning)** | A dose of Tdap during every pregnancy, preferably during the early part of the third trimester (Weeks 27–36). Adults who anticipate having close contact with an infant should be vaccinated with Tdap at least 2 weeks before contact with the infant | A dose of Tdap-IPV during every pregnancy, in the second or third trimester (Weeks 20–36). If the mother did not receive a vaccination during pregnancy, those likely to be in close contact with the infants during their first 6 months of life should also be vaccinated | A dose of Tdap during every pregnancy, preferably early third trimester (Week 28 onwards) or during the second trimester if preterm birth is forthcoming. All other individuals in close contact with newborns and young infants should be vaccinated if they have not received a pertussis vaccination in the last 10 years |
| **Healthcare personnel** | A dose of Tdap for those who have not previously received Tdap and who have direct patient contact;  Td or Tdap every 10 years | A dose of Tdap-IPV at ages 25, 45, and 65 years (unless they received pertussis vaccination within the last 5 years) | A dose of Tdap (or Tdap-IPV if indicated) every 10 years |

^a^ Adults aged beyond 45 years mainly receive Td-IPV; Tdap-IPV to be used in specific cases.
^b^ Adults are to receive Tdap-IPV if indicated.

IPV, inactivated poliovirus vaccine; Td; tetanus-diphtheria vaccine with reduced antigenic dose of diphtheria; Tdap, tetanus-diphtheria-acellular pertussis vaccine with reduced antigenic doses of diphtheria and acellular pertussis.

|  |  |  |  | **Overall** (*N* = 728) | | | **US** (*n* = 354) | | **France** (*n* = 184) | | **Germany** (*n* = 190) |
| --- | --- | --- | --- | --- | --- | --- | --- | --- | --- | --- | --- |
| Immunocompromised patients | | | | |  |  | |  | |  | |
| Patients with COPD | | | | |  |  | |  | |  | |
| Patients with other respiratory conditions | | | | |  |  | |  | |  | |
| Patients with asthma | | | | |  |  | |  | |  | |
| Elderly patients (≥ 65 years old) | | | | |  |  | |  | |  | |
| Smokers | | | | |  |  | |  | |  | |
| Patients facing occupational hazards | | | | |  |  | |  | |  | |
| Patients with diabetes | | | | |  |  | |  | |  | |
| Pregnant women | | | | |  |  | |  | |  | |
| Patients with cardiovascular disease | | | | |  |  | |  | |  | |
| Obese patients | | | | |  |  | |  | |  | |
| Patients with neurologic disorders | | | | |  |  | |  | |  | |

**Fig. 1** Patients considered by physicians to be most at risk of pertussis. Response to the study question ‘To the best of your knowledge, which of the following groups of adult patients are most at risk of pertussis?’

COPD, chronic obstructive pulmonary disease

|  |  |  |  | **Overall** (*N* = 800) | | | **US** (*n* = 400) | | **France** (*n* = 200) | | **Germany** (*n* = 200) |
| --- | --- | --- | --- | --- | --- | --- | --- | --- | --- | --- | --- |
| Patients without medical issues | | | | |  |  | |  | |  | |
| Patients with asthma | | | | |  |  | |  | |  | |
| Patients with COPD | | | | |  |  | |  | |  | |
| Patients with other conditions | | | | |  |  | |  | |  | |

**Fig. 2** Physician’s level of vaccine recommendation in adult patients with and without medical conditions. Response to the study question ‘Approximately to what proportion of adult patients (≥ 18 years old) suffering from asthma and/or COPD do you [US: recommend; France/Germany: prescribe] pertussis-containing booster vaccines in a typical [US/France: month; Germany: quarter]?’

COPD, chronic obstructive pulmonary disease

| **Overall** (*N* = 800) |
| --- |
| **US** (*n* = 400) |
| **France** (*n* = 200) |
| **Germany** (*n* = 200) |

**Fig. 3** Frequency of physician recommendation of pertussis boosters in patients with asthma and/or COPD. Response to the study question ‘Do you currently recommend pertussis-containing booster vaccines to your adult patients with asthma and/or COPD?’

COPD, chronic obstructive pulmonary disease

|  |  |  |  | **Overall** (*N* = 800) | | | **US** (*n* = 400) | | **France** (*n* = 200) | | **Germany** (*n* = 200) |
| --- | --- | --- | --- | --- | --- | --- | --- | --- | --- | --- | --- |
| Patients without medical issues | | | | |  |  | |  | |  | |
| Patients with asthma | | | | |  |  | |  | |  | |
| Patients with COPD | | | | |  |  | |  | |  | |
| Patients with other conditions | | | | |  |  | |  | |  | |

**Fig. 4** Conversion rates in adult patients who received a pertussis vaccination after recommendation from their physician. Response to the study question ‘Out of your adult patients to whom you have [US: recommended; France: prescribed; Germany: prescribed/recommended] a pertussis-containing booster vaccine, what proportion received it?’ To obtain conversion rates, responses were divided by the number of patients the pertussis vaccine was prescribed/recommended to

COPD, chronic obstructive pulmonary disease

| **Overall** (*N* = 800) |
| --- |
| **US** (*n* = 400) |
| **France** (*n* = 200) |
| **Germany** (n = 200) |

**Fig. 5** Impact of the COVID-19 pandemic on physicians’ level of recommendation of pertussis vaccination. Response to the study question ‘To what extent, if any, would you say the COVID-19 pandemic has impacted on your [US: recommendation; France: prescription; Germany: prescription/recommendation] of pertussis-containing booster vaccines?’

|  | **Overall** (*N* = 800) | | **US** (*n* = 400) | | **France** (*n* = 200) | | **Germany** (*n* = 200) |
| --- | --- | --- | --- | --- | --- | --- | --- |
| Vaccine efficacy |  | | | | | | |
| Patients most at risk |  | | | | | | |
| Reminder of vaccination schedule |  | | | | | | |
| Co-administration guidance |  | | | | | | |
| Data on long-term complications of pertussis |  | | | | | | |
| Information related to the disease in adults |  | | | | | | |
| Information on vaccination in specific patient groups (age/high-risk) |  |  | |  | |  | |
| Exceptional cases (pregnancies, allergies, autoimmune diseases) |  | | | | | | |
| Safety information for patients |  | | | | | | |
| Epidemiology data |  | | | | | | |

**Fig. 6** Most interesting topics to physicians in relation to pertussis and pertussis vaccination. Response to the study question ‘What type of information are you most interested to find out about pertussis disease and pertussis-containing booster vaccines for adult patients (≥ 18 years old)?’. Respondents were asked to choose from a list of multiple-choice answers. Respondents were allowed to select more than one answer. Only the top 10 mentions are shown.

# References

1. Centers for Disease Control and Prevention. The Pink Book. Chapter 16: Pertussis. 2022. https://www.cdc.gov/vaccines/pubs/pinkbook/pert.html. Accessed 9 August 2023
2. Havers FP, Moro PL, Hunter P, Hariri S, Bernstein H. Use of tetanus toxoid, reduced diphtheria toxoid, and acellular pertussis vaccines: Updated recommendations of the Advisory Committee on Immunization Practices - United States, 2019. MMWR Morb Mortal Wkly Rep. 2020;69:77-83.
3. Centers for Disease Control and Prevention. Evaluating Revaccination of Healthcare Personnel with Tdap: Factors to Consider. 2022. <https://www.cdc.gov/vaccines/vpd/pertussis/tdap-revac-hcp.html>. Accessed 10 August 2023.
4. Centers for Disease Control and Prevention. Altered immunocompetence. 2023. <https://www.cdc.gov/vaccines/hcp/acip-recs/general-recs/immunocompetence.html>. Accessed 10 August 2023.
5. Ministère de la Santé et de la Prévention. Le calendrier des vaccinations. 2023. <https://sante.gouv.fr/prevention-en-sante/preserver-sa-sante/vaccination/calendrier-vaccinal>. Accessed 10 August 2023.
6. Haute Autorité de santé. Recommandation vaccinale contre la coqueluche chez la femme enceinte. 2022. <https://www.has-sante.fr/jcms/p_3084228/fr/recommandation-vaccinale-contre-la-coqueluche-chez-la-femme-enceinte>. Accessed June 2023.
7. Haut Conseil de la Santé Publique. Vaccination des personnes immunodéprimées ou aspléniques. Recommandations actualisées. 2014. <https://www.hcsp.fr/explore.cgi/avisrapportsdomaine?clefr=504>. Accessed 10 August 2023.
8. Robert Koch Institut. Epidemiologisches Bulletin 4/2023. 2023. <https://www.rki.de/EN/Content/infections/Vaccination/recommandations/04_23_englisch.pdf?__blob=publicationFile>. Accessed 10 August 2023.
9. Laws HJ, Baumann U, Bogdan C, Burchard G, Christopeit M, Hecht J, Heininger U, Hilgendorf I, Kern W, Kling K, et al. Impfen bei Immundefizienz : Anwendungshinweise zu den von der Ständigen Impfkommission empfohlenen Impfungen. (III) Impfen bei hämatologischen und onkologischen Erkrankungen (antineoplastische Therapie, Stammzelltransplantation), Organtransplantation und Asplenie. Bundesgesundheitsblatt Gesundheitsforschung Gesundheitsschutz. 2020;63:588-644.
10. Robert Koch Institut. Epidemiologisches Bulletin 4/2021. 2021. <https://www.rki.de/DE/Content/Infekt/EpidBull/Archiv/2021/Ausgaben/04_21.html>. Accessed 10 August 2023.
